# Costs and Access Barriers to Ondansetron in the US

**DOI:** 10.1001/jamanetworkopen.2024.43978

**Published:** 2024-11-07

**Authors:** Changchuan Jiang, K. Robin Yabroff, Ryan D. Nipp, Xuesong Han, Xin Hu, Joshua M. Liao, Ya-Chen Tina Shih

**Affiliations:** 1 Division of Hematology and Oncology, Department of Internal Medicine, University of Texas Southwestern Medical Center, Dallas; 2 Surveillance and Health Equity Science, American Cancer Society, Atlanta, Georgia; 3 Department of Medicine, University of Oklahoma Health Sciences Center, Oklahoma City; 4 Department of Radiation Oncology, Emory University School of Medicine, Atlanta, Georgia; 5 Division of General Internal Medicine, Department of Internal Medicine, University of Texas Southwestern Medical Center, Dallas; 6 Program in Cancer Health Economics Research, Jonsson Comprehensive Cancer Center, and Department of Radiation Oncology, School of Medicine, University of California, Los Angeles

## Abstract

This cross-sectional study compares the use of management tools and plan-level total costs for oral ondansetron between Part D independent prescription drug plans and Medicare Advantage prescription drug plans.

## Introduction

Ondansetron, the most commonly recommended antinausea drug for patients with cancer, initially faced high list prices, prompting scrutiny and national campaigns supported by the American Society of Clinical Oncology to reduce use.^1,2^ However, the introduction of generic ondansetron in 2006 dramatically lowered costs and increased its value, leading to its inclusion on the World Health Organization’s essential medication list for all health systems globally.^3^

In the US, approximately 80% of Medicare beneficiaries receive prescription drug benefits via Part D independent prescription drug plans (PDPs) or Medicare Advantage prescription drug plans (MAPDs).^4^ These plans may impose management tools, such as prior authorization and quantity limit policies, which can hinder patients’ access to ondansetron.^5^ With MAPDs now covering 50% Medicare beneficiaries and its operational differences from PDPs, this study compared the use of management tools and plan-level total costs for oral ondansetron between these 2 plan types. We further assessed ondansetron’s total cost with comparator prices.

## Methods

The UTSW Human Research Protection Program deemed this cross-sectional study exempt from review and did not require informed consent because it was not human subject research. This study adhered to the STROBE reporting guideline. We used 2023 quarter 3 PDP and MAPD data from the Center for Medicare & Medicaid Services (CMS).^4^ Prior authorization and quantity limit requirements were obtained from the Basic Drugs Formulary File. We focused on 4 common generic ondansetron forms in oncology practice: 4-mg and 8-mg tablets, and 4-mg and 8-mg orally disintegrating tablets (ODT).^2^ To compare across 4 different forms, we examined the mean (SE) cost of 30-day supply (90 dosage units), based on the mean unit cost for each formulation. Comparators were the cash price (without insurance) through the Mark Cuban Cost Plus Drug Company (MCCPDC), a direct-to-consumer pharmacy pricing their drugs with fixed markup to negotiated wholesale prices.^6^ We used *t* test and χ^2^ tests to compare costs and prevalence of prior authorization and quantity limit between PDPs and MAPDs. Two-sided *P* < .05 was statistically significant. All analyses were weighted based on plan-level enrollment data and used SAS version 9.4 (SAS Institute).

## Results

Across 813 PDPs and 3512 MAPDs, all plans covered all 4 ondansetron formulations, with uniform coverage policies across 4 doses and forms. Compared with PDPs, MAPDs used prior authorization and quantity limits more frequently (prior authorization: 90.3% [95% CI, 89.3%-91.3%] vs 71.9% [95% CI, 68.8%-75.0%]; *P* < .001; quantity limits: 22.5% [95% CI, 27.1%-30.1%] vs 16.5% [95% CI, 13.5%-18.6%]; *P* < .001).

Compared with PDPs, MAPDs had lower total costs for ondansetron tablets (4 mg: $24.4 [95% CI, $23.7-$25.2] vs $31.4 [$30.1-$32.7]; *P* < .001; 8 mg: $31.5 [$30.2-$32.9] vs $35.7 [$33.2-$38.2]; *P* < .001), but similar costs for ODTs (4 mg: $46.1 [95% CI, $44.9-$47.3] vs $46.0 [95% CI, $44.6-$47.4]; *P* = .91; 8 mg: $53.2 [$51.7-$54.7] vs $51.4 [$49.2-$53.6]; *P* = .18). Mean costs for both PDPs and MAPDs were significantly higher than MCCPDC prices (all *P* < .001) (Table, Figure).

**Table.** Thirty-Day Cost of Ondansetron Across Medicare Advantage Drug Plans and Independent Part D Prescription Drug Plans^a^

Ondansetron	Total cost, $ (95% CI)^b^		MCCPDC cash price (without insurance), $
	MAPDs (n = 3512)	Part D PDPs (n = 813)	
4-mg Tablet	24.4 (23.7-25.2)	31.4 (30.1-32.7)	9.5
4-mg ODT	46.1 (44.9-47.3)	46.0 (44.6-47.4)	22.1
8-mg Tablet	31.5 (30.2-32.9)	35.7 (33.2-38.2)	14.9
8-mg ODT	53.2 (51.7-54.7)	51.4 (49.2-53.6)	22.1

Abbreviations: MAPDs, Medicare Advantage prescription drug plans; MCCPDC, Mark Cuban Cost Plus Drug Company; ODT, oral disintegrating tablets; PDPs, independent prescription drug plans.

^a^ Data sources: Centers for Medicare & Medicaid Services (CMS) plan enrollment data as of October 2023 and the Quarterly Prescription Drug Plan Formulary, Pharmacy Network, and Pricing Information (2023 Quarter 3). To concentrate on the plans most widely available to the public, we included all PDP and MAPD plans but excluded employer-sponsored and Supplemental Need Plans (including those for dual-eligible individuals) as well as plans with fewer than 10 enrollees per CMS plan enrollment in October 2023.

^b^ Total costs were weighted based on plan-level enrollment of MAPDs and PDPs according to CMS enrollment data October 2023.

**Figure.**
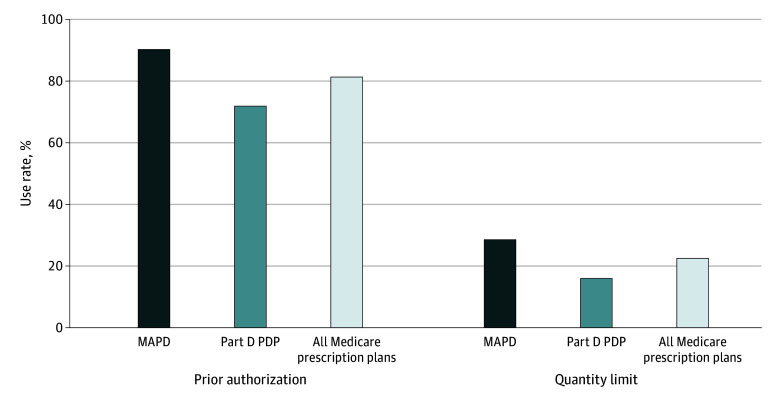
Prior Authorization and Quantity Limits Rates for Ondansetron Across Medicare Prescription Drug Plans^a^ MAPD indicates Medicare Advantage prescription drug plan; PDP, independent prescription drug plan. ^a^All analyzed plans covered all 4 ondansetron formulations, with uniform coverage policies across 4 doses and forms. Analyses were weighted based on plan-level enrollment of MAPDs and PDPs according to Centers for Medicare & Medicaid Services (CMS) enrollment data October 2023. Data sources: CMS plan enrollment data as of October 2023 and the Quarterly Prescription Drug Plan Formulary, Pharmacy Network, and Pricing Information (2023 Quarter 3). To concentrate on the plans most widely available to the public, we included all PDP and MAPD plans but excluded employer-sponsored and Supplemental Need Plans (including those for dual-eligible individuals) as well as plans with fewer than 10 enrollees per CMS plan enrollment in October 2023.

## Discussion

This cross-sectional study found that both PDPs and MAPDs use prior authorization at high rates (over 70%) for ondansetron, an essential, high-value antiemetic for patients with cancer. MAPDs, despite often having lower costs, used prior authorization and quantity limits more frequently than PDPs. Both plan types offer the drug at higher total costs than MCCPDC cash price.

Previous efforts to rationalize antiemetic use led payors and partnering pharmacy benefit managers (PBMs) to impose restrictions for cost control when prices were higher.^1,5^ However, these restrictions persist despite ondansetron’s decreasing cost to payors and PBMs when generic ondansetron became available.^5^ A limitation of this study is the use of plan-level data, preventing us from assessing the association of ondansetron restrictions with individual patient outcomes.

Despite ondansetron’s decreasing costs, most MAPDs and PDPs continue to enforce substantial utilization management, limiting patients’ timely access while increasing clinicians’ administrative burdens. Even with 100% approval rates, prior authorizations can still create access barriers, especially when patients need immediate symptom relief, increasing the risk of avoidable acute care due to nausea and associated downstream costs.^5^ Policymakers must prioritize access to high-value medications to prevent unnecessary delays and cost in care.

## Notes

Supplement.
Data Sharing Statement
