# Supplementary material for: Costs and Access Barriers to Ondansetron in the US
Source: JAMA Netw Open. 2024 Nov 7;7(11):e2443978. doi: 10.1001/jamanetworkopen.2024.43978 (PMC11544490; doi:10.1001/jamanetworkopen.2024.43978)
Supplement: Supplement. — Data Sharing Statement [file jamanetwopen-e2443978-s001.pdf]

Data Sharing Statement

Jiang. Costs and Access Barriers to Ondansetron in the US. *JAMA Netw Open*. Published November 07, 2024. doi:10.1001/jamanetworkopen.2024.43978

Data
Data available: Yes
Data types: Data (not involving human participants), Data dictionary
How to access data: The analysis is based on publically accessible data in CMS PBP file
When available: With publication

Supporting Documents
Document types: None

Additional Information
Who can access the data: The analysis is based on publically accessible data in CMS PBP file. Anyone requesting the data will be accessed.
Types of analyses: For research purpose
Mechanisms of data availability: with investigator support
